# High-throughput gene-expression quantification of grapevine defense responses in the field using microfluidic dynamic arrays

**DOI:** 10.1186/s12864-016-3304-z

**Published:** 2016-11-22

**Authors:** Marie-Cécile Dufour, Noël Magnin, Bernard Dumas, Sophie Vergnes, Marie-France Corio-Costet

**Affiliations:** 1 INRA, UMR Santé et Agroécologie du vignoble (SAVE-1065), CS 20032, ISVV, 33882 Villenave d’Ornon, CEDEX France; 2 Université de Toulouse, UPS, UMR 5546, Laboratoire de Recherche en Sciences Végétales, BP 42617, Auzeville, F-31326 Castanet-Tolosan, France; 3 CNRS, UMR 5546, BP 42617, F-31326 Castanet-Tolosan, France

**Keywords:** BTH, Defense responses, Dynamic array, Elicitation, Fosetyl, Gene expression, Grapevine, Real-time qPCR, Vineyard experiments, *Plasmopara viticola*

## Abstract

**Background:**

The fight against grapevine diseases due to biotrophic pathogens usually requires the massive use of chemical fungicides with harmful environmental effects. An alternative strategy could be the use of compounds able to stimulate plant immune responses which significantly limit the development of pathogens in laboratory conditions. However, the efficiency of this strategy *in natura* is still insufficient to be included in pest management programs. To understand and to improve the mode of action of plant defense stimulators in the field, it is essential to develop reliable tools that describe the resistance status of the plant upon treatment.

**Results:**

We have developed a pioneering tool (“NeoViGen96” chip) based on a microfluidic dynamic array platform allowing the expression profiling of 85 defense-related grapevine genes in 90 cDNA preparations in a 4 h single run. Two defense inducers, benzothiadiazole (BTH) and fosetyl-aluminum (FOS), have been tested *in natura* using the “NeoViGen96” chip as well as their efficacy against downy mildew.

BTH-induced grapevine resistance is accompanied by the induction of PR protein genes (*PR1*, *PR2* and *PR3*), genes coding key enzymes in the phenylpropanoid pathway (*PAL* and *STS*), a *GST* gene coding an enzyme involved in the redox status and an *ACC* gene involved in the ethylene pathway.

FOS, a phosphonate known to possess a toxic activity against pathogens and an inducing effect on defense genes provided a better grapevine protection than BTH. Its mode of action was probably strictly due to its fungicide effect at high concentrations because treatment did not induce significant change in the expression level of selected defense-related genes.

**Conclusions:**

The NeoViGen96” chip assesses the effectiveness of plant defense inducers on grapevine in vineyard with an excellent reproducibility. A single run with this system (4 h and 1,500 €), corresponds to 180 qPCR plates with conventional Q-PCR assays (Stragene system, 270 h and 9,000 €) thus a throughput 60–70 times higher and 6 times cheaper. Grapevine responses after BTH elicitation in the vineyard were similar to those obtained in laboratory conditions, whereas our results suggest that the protective effect of FOS against downy mildew in the vineyard was only due to its fungicide activity since no activity on plant defense genes was observed. This tool provides better understanding of how the grapevine replies to elicitation in its natural environment and how the elicitor potential can be used to reduce chemical fungicide inputs.

**Electronic supplementary material:**

The online version of this article (doi:10.1186/s12864-016-3304-z) contains supplementary material, which is available to authorized users.

## Background

The grapevine cultivated in Europe (*Vitis vinifera*) is subject to diseases due to many bioagressors, notably obligate fungal and oomycete parasites such as powdery mildew (*Erysiphe necator*) and downy mildew (*Plasmopara viticola*). Control of epidemics requires numerous chemical treatments with harmful effects on the environment and human health. In addition to plant breeding and biological control, the use of plant defense stimulators (elicitors) could be a promising alternative.

Usually acting on the plant and not directly on the pathogen, elicitors induce a multi-factorial plant resistance which is probably more difficult to overcome by the pathogen than protection provided by an single-site fungicide [1]. There are a wide variety of abiotic or biotic elicitors of animal, plant, fungal or bacterial origin [2]. In recent years, considerable progresses have been made to identify the mode of action of elicitors on various plant models and to identify the genes involved in defense responses [3]. The induced immunity activates a wide variety of defense mechanisms that involve passive defense mechanisms [4] that restrict the entry or spread of the pathogen in the plant, but also active defense mechanisms that prevent the development of the pathogen by confining it to the site of infection or causing its death. The most common early cellular responses are mechanisms of ion flux changes, production of reactive oxygen species (ROS) and phosphorylation mechanisms/dephosphorylation (mitogen -activated protein kinase or MAPKKK, MAPKK and MAPK [5–7]. After these early steps, some secondary metabolic pathways are stimulated and allow the generalization of the response to the whole plant, while systemic acquired resistance (SAR) is being established [8, 9]. SAR requires systemic movement of signals from the infected tissue to healthy tissue. Molecules such as salicylic acid (SA), jasmonic acid (JA), ethylene (ET), systemin and even hydrogen peroxide, which are involved in the different signaling pathways, are activated in response to elicitation [10–13]. They rapidly accumulate in the cell and allow the defense genes to be expressed. Regulating defenses by SA, JA/ET is complex and to date these signaling pathways have appeared to interact with each other [14, 15]. Following the defense reaction, the intracellular signaling pathways in plants converge towards the production of active forms of oxygen and hormones (SA, JA, ET or ABA). Final steps correspond to the induction of defense genes, the production of secondary metabolites (phytoalexins, PR-proteins) and the strengthening of cell walls, which all contribute to stopping the development of the pathogen [2, 3, 5]. However, despite considerable progress in understanding the activity of elicitors and their reproducible effects in controlled laboratory conditions, their application *in natura* on crops such as grapevine has been rather disappointing [2].

In view of this situation, greater insight is needed into grapevine immune responses in relation to the genetic background of the plant, pathogen diversity and environmental conditions. Preliminary studies in our laboratory allowed us to select potential elicitors with a defined chemical composition, and which have stable reproducible efficiency under controlled conditions against the two major pathogens of grape: powdery mildew (*Erysiphe necator*) and downy mildew (*Plasmopara viticola*). We focused particularly on phosphonates and benzothiadiazole (BTH or acibenzolar-S-methyl (ASM)), which are already known as stimulators of plant defenses [16–23]. BTH has been shown to be effective against a broad spectrum of pathogens in various plants [19–30], with no direct antifungal activity, thereby clearly establishing its role as an inducer that is dependent on the salicylic acid (SA) pathway [31]. According to its mode of action, BTH is classified by FRAC (Fungicide Resistance Action Committee, http://www.frac.info/) in P1 group, no reporting any resistance phenomenon until now. Fosetyl-aluminum (FOS, [aluminum tris (ethyl phosphonate)]), is a phosphonate used against diseases caused by oomycetes [32]. The mode of action of FOS is multi-site which avoids resistance phenomenons, being classified by FRAC in 33 group. It is remarkable that according to FRAC, few resistance cases have been reported in few pathogens after more than 30 years of utilization. It has a complex mode of action with a direct effect on pathogens at high doses as well as an indirect activity thanks to enhanced plant defense responses at low doses [33]. Phosphonates have been widely studied for their role as phytoalexin inducer [33–37].

To monitor the activity of these compounds in the field, it is possible to assess their defense inducer effect by analyzing the expression of a significant number of marker genes involved in the defense process of grapevine. Recently, two molecular diagnostic tools were designed that provide information about the defense status of grapevine: “qPFD” (quantitative RT-PCR microplate/DNA chip low density) which was first developed on the apple scab model (*Malus domestica*/*Venturia inaequalis*) and extended to grapevine and evaluates a set of nine groups representing 28 target genes (patent INRA WO 2011/161388, CT/FR2011/051470 - INRA Angers - Brisset MN) [38]; and “BioMolChem”chip which is based on 20 marker genes highly involved in grapevine defense mechanisms [26]. However, a more accurate diagnostic tool would certainly require a larger number of defense markers since genomic analyses have shown that induction of the plant immune system is linked to changes in the expression of thousands of genes [39]. Recently, considerable progress has been made in the development of automated platforms that enable the high-throughput analysis of gene expression by Q-PCR [40], notably involving microfluidic chips.

Here, we describe the construction of a new “NeoVigen96” chip allowing the detection of 85 defense markers and 11 genes used for standardization of expression (constitutively expressed genes) on 95 cDNA preparations in a single run. The chip was used to study the inducer activity of BTH and FOS in leaves collected in the field.

The general idea of this work is not to demonstrate that these BTH and FOS applications should be used as they are performed in this article in the context of conventional programs to protect the grapevine, but to demonstrate that it is possible to test the effects of potential elicitor products on grapevine defense responses with the Fluidigm tool. The resulting data provide better understanding of grapevine defense status with a view to optimizing the potential of plant defense elicitors.

## Results and discussion

### “NeoViGen 96” chip conception and validation

Induction of plant immunity implements molecular signaling cascades that ultimately lead to different levels of mechanical and chemical protection. Typically, this inducible resistance system is controlled by phytohormones such as salicylic acid (SA) [8], jasmonic acid and ethylene, leading to the coordinated accumulation of pathogenesis-related proteins (PR proteins), the production of phytoalexins and the reinforcement of plant cell walls [3].

We used various strategies in order to obtain the most recent molecular data and find homologs to the already known responsive gene sequences and find new targets. An additional file shows the origin of the sequences and/or references used to find new candidate genes involved in grapevine defenses (Additional file 1). The strategy combines two approaches : the first was based on the comparison of new grapevine genomic data with known grapevine sequences previously selected from pathogen-related studies to which were added genes deployed in two recently developed molecular diagnostic tools (“grapevine - qPFD”, patent INRA FR 1055042/WO/2011/161388, CT/FR2011/051470 - INRA Angers - Brisset MN, [38] and “BioMolChem” chip, [26]). The sequences used in RT-qPCR were blasted against the most recent *Vitis vinifera* sequences (taxid = 29,760, [41]) using the Blast resource from the National Center for Biotechnology Information (https://blast.ncbi.nlm.nih.gov/Blast.cgi). Nucleotide sequences were used and results were manually curated to find homologs to the original sequences used in the RT-qPCR experiments. Homology was confirmed by aligning selected sequences with Clustal [42] and generating phylogenetic trees. Once recovered, the sequences to be included in the Fluidigm protocol were subjected to the primer-blast program [43] for specific primer design.

The second strategy combined with the first involved the recovery of sequences from the model organism *A. thaliana* in the Genevestigator database [44]. Micro-array experiments involving foliar fungal pathogens deposited in Genevestigator were selected and the most differentially expressed plant sequences between control and treated samples were recovered. These sequences (*N* = 273) were identified on the NCBI website and aligned against the most recent *Vitis vinifera* sequences using the NCBI Blast resource. The mean percentage of *Vitis vinifera* protein sequence homology/ortology with those of *Arabidopsis thaliana* and/or *Malus domestica* was 53%, between 16 and 99 (Additional file 2).

**Fig. 1:**
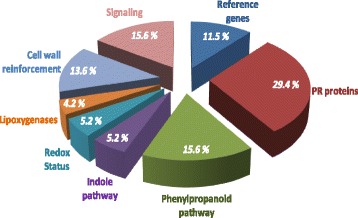
Function of genes analyzed using the “NeoViGen96” chip The “NeoViGen96” chip included genes coding for PR proteins (*n* = 28), enzymes involved in the synthesis of secondary metabolites (phenylpropanoids, terpenoids, *N* = 15; and indole compounds (*N* = 5), in the oxido-reduction system (*N* = 5), in the ethylene or oxylipin/JA pathways (*N* = 4), cell wall reinforcement (*N* = 13) and other proteins involved in pathogen detection signaling and transcription signaling (*N* = 15)

The combination of the two strategies gave rise to 96 new *Vitis* sequences included in the Fluidigm protocol (Tables 1 and 2). The gene set included reference genes (*N* = 11), PR proteins (*N* = 28), some genes involved in secondary metabolites (phenylpropanoids, *N* = 15) and indole pathway (*N* = 5), others involved in the oxido-reduction system (*N* = 5), in the ethylene or oxylipine/JA pathways (*N* = 4), cell wall reinforcement (*N* = 13) and others involved in pathogen detection-signaling and transcription signaling (*N* = 15; Fig. 1).

**Table 1:** Genes used in “BioMolChem” chip that were analyzed in the Stratagene Mx3005P qPCR system, classified according to functions and pathways

	Defense-related genes	Names	N° accession GeneBank	Forward primer (5′-3′)	Reverse primer (5′-3′)
*Reference gene*	γ-chain of Elongation Factor 1	*EF1γ*	AF176496	GAAGGTTGACCTCTCGGATG	AGAGCCTCTCCCTCAAAAGG
*PR proteins*	PR1 Unknown function	*PR1*	AJ536326	CCCAGAACTCTCCACAGGAC	GCAGCTACAGTGTCGTTCCA
	Beta-1,3-glucanase (PR2)	*GLU*	AF239617	GGGGAGATGTGAGGGGTTAT	TGCAGTGAACAAAGCGTAGG
	Endochitinase (Chitinase IV - PR3)	*CHIT4a*	U97521.1	TATCCATGTGTCTCCGGTCA	TGAATCCAATGCTGTTTCCA
	proteinase inhibitor (PR6)	*PIN*	XM_002284418	ACGAAAACGGCATCGTAATC	TCTTACTGGGGCACCATTTC
	Chitinase III - (PR8)	*CHIT3*	Z68123	AATGATGCCCAAAACGTAGC	ATAAGGCTCGAGCAAGGTCA
	PR protein - class 10 (PR10)	*PR10*	AJ291705	GCTCAAAGTGGTGGCTTCTC	CTCTACATCGCCCTTGGTGT
	Polygalacturonase Inhibiting Protein	*PGIP*	XM_002263487.1	GAGCGATGCCACCCCAGTGA	CCGTTGAGTCGGACGCTCGAC
*Phenylpropanoid pathways*	Phenylalanine ammonia lyase	*PAL*	X75967	ACAACAATGGACTGCCATCA	CACTTTCGACATGGTTGGTG
	Stilbene synthase	*STS*	X76892.1	ATCGAAGATCACCCACCTTG	CTTAGCGGTTCGAAGGACAG
	Chalcone Synthase	*CHS*	X75969.1	CCAACAATGGTGTCAGTTGC	CTCGGTCATGTGCTCACTGT
	Chalcone Isomerase	*CHI*	X75963	AGAAGCCAAAGCCATTGAGA	CCAAGGGGAGAATGAGTGAA
	Anthocyanidine synthase	*LDOX*	X75966	TGGTGGGATGGAAGAGCTAC	CCCACTTGCCCTCATAGAAA
	Flavanone-3-hydroxylase	*F3H*	X75965.1	TGACTCGCTCTCTTCAAGCA	CACCTTGGGACGTTCATCTT
*Indole pathways*	Antranilate Synthase	*ANTS*	XM 002281597	AAAAATCCAAGAGGGGTGCT	AAGCTTCTCCGATGCACTGT
	Chorismate mutase	*CHORM*	FJ604854	TCATTGAGAGGGCCAAATTC	AGGAGGCAGAAAAAGCATCA
	Chorismate Synthase	*CHORS*	FJ604855	GCCTTCACATGCAGATGCTA	CTGCAACTCTCCCAATGGTT
*Redox status*	Glutathione S-transferase	*GST1*	AY156048.1	GGGATCTCAAAGGCAAAACA	AAAAGGGCTTGCGGAGTAAT
*Lipoxygenases*	Lipoxygenase-9	*LOX9*	AY159556	GACAAGAAGGACGAGCCTTG	CATAAGGGTACTGCCCGAAA
*Signaling*	1-aminocyclopropane, 1-carboxylic oxidase	*ACC*	AF424611	GAAGGCCTTTTACGGGTCTC	CCAGCATCAGTGTGTGCTCT
*Cell Wall Reinforcement*	Glycosyl transferase	*CAGT*	XM02273320.1	TCGGAAGGGAATGCAATAAG	TGTAGGAGGAACCACCCTTG
	Callose Synthase	*CALS*	AJ430780.1	TGGAAATGCAATTCAAACGA	CGAATGCCATGTCTGTATGG
	Lignin-forming peroxidase	*PER*	XM_002274762.1	TAAGCGCCACAAGAACACTG	GGACCTCCTTGTTGAGTCCA

**Table 2:** Genes used in “NeoVigen96” chip that were analyzed in the Biomark HD system, classified according to functions and pathways

Gene Functions		Gene Names	N° accession	Primer Sens - 5′ > 3′ (F)	Primer AntiSens - 5′ > 3′ (R)	PCR efficacies
Reference genes	Elongation factor1 chain γ	*EF1γ* ^a^	*AF176496*	GAAGGTTGACCTCTCGGATG	AGAGCCTCTCCCTCAAAAGG	1.09
	Serine/threonine-protein phosphatase 2A	*PP2A*	XM_002276144	TCCGGCGGCTCTCGACGATT	TTCGCGTGCTCAACACCTCCG	0.97
	SAND family protein (endocytosis)	*SAND*	XM_002285134	GCCCCACAGCCAAACCCCTC	ACGATCCGTTTGCGACCCCG	1.05
	Pentatricopeptide repeat-containing protein	*Unknown*	XM_002274855	TGGTGAACTTGAGGCTGCAAGGG	ACCATTTGGGGAGTAGCCCTTCCTC	1.01
	Ubiquitin Conjugating Enzyme 9	*UBC9*	XM_002274238	TCCTCCTGACAGTCCATATGCTGGT	GGGCTGGGCTCCACTGCTCC	1.00
	TIP41-like protein	*TIP41*	XM_002270674	CAGCGGGCAGCGATCGAAGA	CATTTCCGCTCCGGCAGCCTT	1.05
	Catalytic thioredoxin-like protein 4A	*THIORYLS8*	XM_002283586	TCACTCTGGATGGGCCGTCG	TCCCAATCGTGGCCGAACCG	1.14
	Tubulin alpha	*TuA*	XM_002285685.1	GTCGGCGCTGAAGGTGTGGA	GAGGTGGCGGGCAAACCCTC	0.97
	Tubulin beta	*βTub*	*XM_002275270.1*	TGAACCACTTGATCTCTGCGACTA	CAGCTTGCGGAGGTCTGAGT	0.99
		*TuB*	XM_02275270.1	CGCCACCCGAGTCTCACTGC	CACACCGTGCTCGTCGCAGA	1.04
	Glyceraldehyde 3-phosphate dehydrogenase	*GADPH*	XM_002263109.1	GAAATCAACGGCCCAGCGCG	CCGGTGGATACTGGGGCGGA	0.83
PR protein	PR1 Unknown function	*PR1* ^a^	AJ536326	CCCAGAACTCTCCACAGGAC	GCAGCTACAGTGTCGTTCCA	1.00
		*PR1 bis*	XM_002273752.1	GGGGTTGTGTAGGAGTCCATTAGCA	TGGGCACAGCAGATGTGAGCT	1.13
	Beta-1,3-glucanase	*GLU* ^a^	AF239617	GGGGAGATGTGAGGGGTTAT	TGCAGTGAACAAAGCGTAGG	1.18
		*PR2*	XM_002277475	CAACTTGCCACCGCCAGGGC	AGGGCTTGGAGAGCAGCTTGG	0.96
	Endochitinase (Chitinase type I, II, IV,, VI and VII)	*PR3*	U97522.1	ACTACGGCGCTGCTGGAAACA	TGGCACCGAAACCTTGGCTTAG	1.16
		*CHIT4a* ^a^	U97521.1	TATCCATGTGTCTCCGGTCA	TGAATCCAATGCTGTTTCCA	1.14
	Chitin binding Chitinases type I, II	*PR4*	XM_002264684.1	CCCAGAGCGCCAGCAATGTGA	TTGCTGCGCCATGCCAAGGG	0.95
		*PR4bis*	XM_002264611.1	TGGCTACTGCGGAACAACGGC	CAAGTGGCGCAGTAGGCGCT	1.02
	Thaumatin-like/Osmotin	*PR5*	XM_002282928.1	GGAGGCAATGGTTTCCACCTTGGG	ACTTGGACGGGACCATAGAGGTTAG	0.99
		*PR5bis*	XM_002282874.1	CCCCGGCACCACCAATGCTC	TGGGGGAGAACCGTAGCCCTG	1.18
	Proteinase inhibitor	*PIN* ^a^	XM_002284418	ACGAAAACGGCATCGTAATC	TCTTACTGGGGCACCATTTC	1.23
		*PR6*	XM_002277772	TGGGAAGCAGGCTTGGCCTGA	ACCTGGCTCTCACCGAAGGG	0.99
		*PR6bis*	XM_002280597.1	GCCAGAGCTGGTGGGCGTAC	AGGCGCCATACTCACGATGCC	1.10
	Subtilisin-like endoprotease	*PR7*	XM_002275435.2	TGCTCCCAATCATGGTGGCTGT	TGAAGACTCTGCGGTGTGTCCT	1.01
		*PR7 bis*	XM_002275435.1	CGTTAAGCAGCTGGAAAGGAGCA	TCCTCCGTCAGTCTGGCTGCAA	1.04
	Chitinase type III	*CHIT3* ^a^	Z68123	AATGATGCCCAAAACGTAGC	ATAAGGCTCGAGCAAGGTCA	0.98
		*PR8*	XM_002276329	GCAACAAAGCTCAATGGCACACCAC	CAGCCAAGCTGCCCTCGTTCC	0.96
	Lignin-forming peroxidase	*POX* ^a^	XM_002285687.1	ACTGCACCAAGAAAGAGCACCAG	AGCTGTGCATGTGCCATCCCC	1.14
		*PR9-b*	XM_002285613.1	AGCGAGCGAGAAAGACGCGA	GAGACGACGCCTGGGCAGAC	0.91
	Ribonuclease-like	*PR10* ^a^	*AJ291705*	GCTCAAAGTGGTGGCTTCTC	CTCTACATCGCCCTTGGTGT	0.99
	Chitinase type I	*PR11*	XM_002270543.1	CTCCACTGCGCAAACCGTGGT	TTTGCGTTTTCGGAGGAAATCGTGA	1.10
	Defensin	*PR12*	XM_002281153	GTGCAAGAACTGGGAGGGTGCC	GCAGAAGCATGCAACTCCCGGG	0.88
	Lipid Transfer Protein	*PR14*	XM_002271080	ACAGTTGATCGCCAGGCCGC	GCCCGGAAGCCCACTTGCAA	1.18
		*PR 14bis*	XM_002270934	CGCCACCACACAAGACCGCA	AGGGAGGCCAGCAGCCAGAC	1.01
	Germin-like Protein- Oxalate oxidase	*PR15*		GTTTCCTGGCCCTCATGGAATTGGC	GTGTCCTGCAGTGGGCTTGGA	1.19
		*PR15bis*	XM_002284176.1	GCCATGGCAGATGATTTCTT	TGCAATTTGGGCAACATTTA	0.87
	Polygalacturonase Inhibiting Protein	*PGIP* ^a^	XM_002263487.1	GAGCGATGCCACCCCAGTGA	CCGTTGAGTCGGACGCTCGAC	0.96
Secondary metabolites biosynthesis	Phenylalanine ammonialyase	*PAL* ^a^	X75967	ACAACAATGGACTGCCATCA	CACTTTCGACATGGTTGGTG	1.10
	Stilbene synthase (resveratrol synthase)	*STS* ^a^	X76892.1	ATCGAAGATCACCCACCTTG	CTTAGCGGTTCGAAGGACAG	0.95
	Resveratrol O-methyl-transferases	*ROMT*	FM178870	TGCCTCTAGGCTCCTTCTAA	TTTGAAACCAAGCACTCAGA	0.96
		*ROMT2*	XM_002281445.1	TCCACACTGCTTACGAGCGGT	CAACCCCGCAAATACGCCCTGG	0.99
	Chalcone Synthase	*CHS* ^a^	X75969.1	CCAACAATGGTGTCAGTTGC	CTCGGTCATGTGCTCACTGT	1.01
		*CHS2*	XM_002276885.1	GCATTTTCCGACGAAGTTCACACTG	GTGCCGATGGCCAGAACCGT	1.04
	Chalcone Isomerase	*CHI* ^a^	*X75963*	AGAAGCCAAAGCCATTGAGA	CCAAGGGGAGAATGAGTGAA	1.04
		*CHI2*	XM_002280122	TGTGGGCCATCTGCAACCATGG	GCACTCTCTAGCTGCACCCCG	1.12
	Dihydro Flavonol Reductase	*DFR*	XM_002281822.1	GGCCACCGTTCGCGATCCAA	GAAGACGCCGGTGCAGCCTT	1.05
	Anthocyanidine synthase	*LDOX* ^a^	X75966	TGGTGGGATGGAAGAGCTAC	CCCACTTGCCCTCATAGAAA	0.84
	Polyphenol Oxidase	*PPO*	XM_002275806.1	GGTCCCTCGTTATGGGGCCGA	CCTGGATGGAAATCAGGGCGCC	0.98
	3-hydroxy-3-methylglutaryl Coenzyme A reductase	*HMGR*	XM_002275791.1	AACGCACACTCCGCTCCACG	GCGGCGGCGATCTTCATCGA	0.93
	Farnesyl Pyrophosphate Synthase	*FPPS*	XM_002272605.1	TCGCCAATGGGTCGAGCGTA	TGCCTGCCTTGCAGCAACTTGT	1.03
	(E,E)-alpha-farnesene synthase	*FAR*	XM_002281343.1	GCCATGGCACTCCACCTCTCCTAA	AGGCGGGCTGGTAATGCGCT	0.99
		*FAR2*	XM_002264969.1	TTGCGAGGCAGAAGCTGGCC	TTTGGCCCACGAAAGGCGGG	1.08
	Flavanone-3-hydroxylase	*F3H* ^a^	*X75965.1*	TGACTCGCTCTCTTCAAGCA	CACCTTGGGACGTTCATCTT	0.87
		*F3H bis*	*XM_002275553.1*	TCCAGCCCGTGGAAGGAGCA	TGCTCAGATACTGCCCACCCAA	0.96
	Carboxylesterase	*HSR-203 J*	XM_002285050.1	TGGAGGAAACATCGTTCACA	CCTGGACAATTCTGCCATCT	1.02
Indole biosynthesis	Antranilate Synthase	*ANTS* ^a^	*XM 002281597*	AAAAATCCAAGAGGGGTGCT	AAGCTTCTCCGATGCACTGT	0.84
	Chorismate mutase	*CHORM* ^a^	*FJ604854*	TCATTGAGAGGGCCAAATTC	AGGAGGCAGAAAAAGCATCA	1.05
		*CHORM2*	XM_002284083.1	GGCAAGTTCGTGGCAGAGGCA	GCCGCTGGCTGTCTTGTGCT	1.11
	Chorismate Synthase	*CHORS* ^a^	*FJ604855*	GCCTTCACATGCAGATGCTA	CTGCAACTCTCCCAATGGTT	1.00
		*CHORS2*	XM_002282263	TGTTATGGCGCGCGGTGACT	GCAGCTCTGGCCAGTTGAGCT	1.11
Redox status	Glutathione S-transferase	*GST1* ^a^	AY156048.1	GGGATCTCAAAGGCAAAACA	AAAAGGGCTTGCGGAGTAAT	0.99
		*GST2*	AY156049	CATGAAGGCCGGCCAGCACA	CGCGAAGAATTCGCTCTGGCCA	0.84
		*GST3*	XM_002283178	TGTTTGGCCGCAAACGGGGT	TCCCCAGCCAGGTACTTGCTCT	1.06
		*GST4*	XM_002271673	AGCTGGAATGGCGCACTTGGT	TGGAAAGGTGCATACATGGCCACG	1.00
		*GST5*	XM_002283173	CCTTGAGCTCTACCCTGCCCCA	AGCAGCCAGCCCTAGACATGGA	0.86
Oxylipines	Lipoxygenase 13	*LOX2*	XM_002285538	AAACCGTGCATTCCCGGCCC	GGCAGGGACGTAGCCAACCC	1.02
		*LOX3*	XM_002284499.2	GGACCGGGTTCATGAGCTGTTGG	TGAATGCAGACTCGCCAGCGGT	1.09
		*LOX4*	XM_002280615.1	CCACAAGCGAAGGCGGGCTT	AGCAATGTGCATTTCAGCCATCGA	1.10
	Lipoxygenase 9	*LOX9* ^a^	AY159556	GACAAGAAGGACGAGCCTTG	CATAAGGGTACTGCCCGAAA	0.92
Cell wall reinforcement	Alliinase	*Alli*	XM_002265837.1	CGGCTCAGCCTCATCAAGACCC	GGCATGCATGTCATCTTCCTCAGCC	1.04
		*Alli2*	XM_002266017.1	AGCCCTTCTGGATGCAGCATGC	TGTAGCTTGCGGATGAGCTTCACT	1.00
	Ascorbate peroxidase	*APOX*	XM_002284731.1	AGCTCAGAGGCCTCATCGCTGA	TACCGGCAGAGTGCCATGCG	1.07
		*APOX2*	XM_002278245.1	TCGAAGCTCAGCAGACGCCG	ACGTCCCCGCATCATGCCAC	1.14
	Glycosyl transferase (Coniferyl alcohol glucosyl transferase)	*CAGT* ^a^	*XM02273320.2*	TCGGAAGGGAATGCAATAAG	TGTAGGAGGAACCACCCTTG	0.81
		*CAGT2*	XM_002276999.1	TGTTCATGAGGGCTGCGCCG	CACCAGGCAGCTCACTGGCC	1.00
	Callose Synthase	*CALS* ^a^	AJ430780.1	TGGAAATGCAATTCAAACGA	CGAATGCCATGTCTGTATGG	0.86
		*CALS2*	XM_002283262 (CS-like 12)	ATGGCGTCCAGAAGCGGCTC	GCCGTCTGTGTCCGCGTGAT	0.86
		*CALS3*	XM_002285608.1 (CS-like-10)	GCAGCAGATTGCCACTGCCCA	AGGCAGAATGAGGTGCTCGCC	1.14
	Lignin-forming peroxidase	*PER* ^a^	XM_002274762.1	TAAGCGCCACAAGAACACTG	GGACCTCCTTGTTGAGTCCA	0.93
	Pectin methyl esterase	*PECT1*	XM_002275783.2	TGTTGGCCTCGAGGAGAGGGG	GGGAGGCCTACAGACCAAAAGTCA	1.18
		*PECT2*	XM_002283905.1	GGGTTGCGCCCTGAGGACAC	CAATCACCCGAGCCGCCTGG	1.08
	CAD Cinnamyl alcohol dehydrogenase	*CAD*	XM_002285332.1	AGTCCGATTGGAAGACGGCAGT	TGCCCCTGTCACACACACCA	1.05
		*CAD2*	XM_002268086.1	TCCGGGTATCCCAGGAGAAAGCA	TCCACGGTATCCTTCATGCTCACC	1.07
Signaling	SA Methyl Transferase	*SAMT1*	XM_002262982.1	AATCCTTGCCCAAGTTCCAG	GAGACAACCATTGGAGACTG	1.16
	Allene Oxide Synthase	*AOS1*	XM_002281190.1	TTATGGCTTGCCCTTCTTTGG	ATGGAGTCGAGGAGGACGAT	0.94
	Lipase 3/enhanced disease susceptibility 1	*EDS1a*	XM_002281059.1	CAGGTCACAGCCTGGGTGCG	TCGGGCGGGACGATCTCGTT	1.01
		*EDS1b*	XM_002281871.1	GGAGACGGGGCTGAACGTGC	CCATCGCCGGCACTTGCTCA	0.89
		*EDS1c*	XM_002275822.1	CCAGCACTGCTTGCAGGCGT	TGCTGTGTTCCTGAGTGCCCC	1.04
	Transcription Factors	*WRKY1*	AY585679.1	GGAAATATGGGCAGAAAGCA	ATCTTTTGAGAGGCGTTGGA	1.00
		*WRKY2*	AY596466	AGAGGCAAGGCGATGTAGAA	CTGGGGAACAAGCCTTCATA	1.01
	JAR = Jasmonate-resistant 1	*JAR*	XM_002283193.1	GCAACGGGGCACGACTACTGT	GCCGTGGCGGTGCAAGTACT	0.89
		*JAR2*	XM_002280702.1	CCGAAGTGCTGGCCCCAGAG	AACGCTCACTTCGCCGCTGA	1.00
		*JAR3*	XM_002268242.1	GGAGCAATGCTGCTCCACAGTGG	GGCGTCGAATGTGCCAGGCT	1.01
	ACCO 1	*ACO1*	XM_002273394.1	GCCGGTTTGAAGTTCCAGGCCA	ACTCAAACTGTGGCAATGGGACCC	1.06
		*ACO1b*	XM_002275305.1/XM_002275284.1	CGAGCCCACACTGATGCCGG	TTGAGGAGCTGGAGGCCGCT	1.00
		*ACC* ^a^	AF424611	GAAGGCCTTTTACGGGTCTC	CCAGCATCAGTGTGTGCTCT	0.86
	EIN3-Binding F Box Protein 1	*EIN3*	XM_002285090.1	TTGGCTCTGAACGCGTCCGA	CCCCGGGGCAGAAGGCATCA	0.88
		*EIN3bis*	XM_02285213.1	CCTCGCAAGCGGTCTCGCAT	TGGAGACCCGAGCGCAGGAG	0.98

^a^Genes also included in the “BioMolChem” chip

We verified that the qPCR was specific for each primer set (appropriate specific target) by checking the appropriate size of the amplified product on agarose gel (not shown) and obtained a single peak in the melting curve after each qPCR run. We also checked that the PCR efficiencies for each primer set were similar (0.8–1.2), thereby allowing us to simplify Pfaffl’s model formula for calculating relative expression [45] with 2^- ΔΔCq^ (data not shown).

### Method sensitivities

**Fig. 2:**
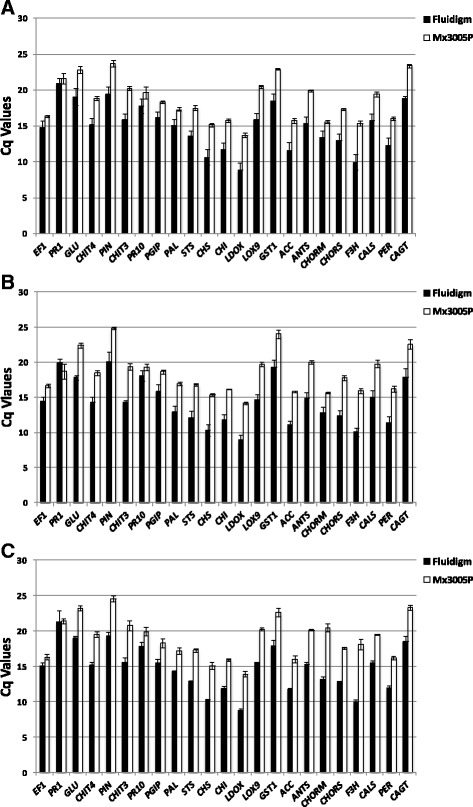
Cq value comparisons using 96.96 dynamic array and Stratagene Mx3005P. cDNAs were synthesized using polydT_(15)_ primers and 10 μg of total RNA from leaves untreated (**a**) or treated with BTH (**b**), Fosetyl-Al (**c**). Bars represent the means of Ct values from three biological replicates. Open bars: Stratagene MX3005P system and closed bars: 96.96 Fluidigm dynamic array

The Cq values obtained on a subset of 23 genes were compared for the same samples in two real-time PCR systems: the Stratagene Mx3005P and the Biomark HD, a Fluidigm® integrated fluidic circuits (IFCs) by automating PCR reactions in nanoliter volumes [46]. Twenty-two out of 23 mRNAs exhibited lower Cq values in the Fluidigm dynamic array than those obtained with the Stratagene MX3005P (15.60 ± 0.42 for the 96.96 dynamic array and 19.54 ± 0.42 for the Stratagene, mean difference, 3.96 ± 0.17), suggesting that the microfluidic technology exhibited a greater sensitivity than the Stratagene while the amounts of cDNA used in this technique were 70–150 times lower (Fig. 2a, b and c).

### Comparison of mRNA expression between 96.96 dynamic array and Stratagene Mx3005P

**Fig. 3:**
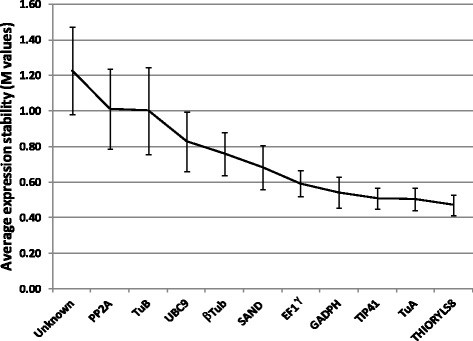
Expression stability mean values (*M*-values). *M* values of 11 endogenous control genes in leaf samples using the principles and formulas described by Vandesompele. [47] Values are means ± SD of 15 independent microfluidic dynamic arrays

Eleven genes were selected to test the stability of their expression in all of the samples studied (control and treated, Table 2) so as to identify constitutive markers that could be used to normalize qPCR results. Multiple-gene normalization was based on the principles and formulas described by Vandesompele [47]. Genes were considered as stable when their *M* values were less than 1.5 (Fig. 3). In our study, all selected genes were considered stable (M mean value = 0.73 ± 0.25, Fig. 3) and multiple-gene normalization was performed with the geometric mean of all reference genes as an accurate normalization factor. The most stable genes in the samples studied were *THIORYL58*, *TuA*, *TIP41, GAPDH* and *EF1γ* (Fig. 3) with *M* values between 0.46 and 0.68 (0.52 ± 0.04). The optimal number of genes required for normalization of RT-PCR data was fixed with these 5 genes for subsequent experiments with a second “NeoVigen 96” chip version.

**Fig. 4:**
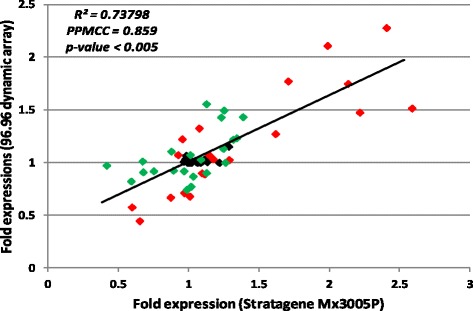
Correlation scatter plots of fold expression of “BioMolChem” chip genes (*n* = 24) using Stratagene or Biomark HD systems obtained with three replicates of leaves treated with BTH (*red*), with FOS (*green*) or untreated (*black*). Correlation of fold expression obtained by Stratagene MX3005P system (x axis) and microfluidic dynamic array (y axis). *R* ^*2*^ = coefficient of correlation of the simple linear regression, PPMCC = Pearson product–moment correlation coefficient (Pearson’s correlation). Significant correlation was determined at a level of *p*-value < 0.05

We compared fold-change expression of defense-related gene in the same samples measured by the 96.96 dynamic array with those obtained from the “BioMolChem” chip with the Stratagene Mx3005P (Fig. 4). Fold change comparisons were similar between the two platforms, which indicated a perfect significant correlation between the two technologies (*R* ^*2*^ = 0.737 and Pearson’s correlation (PPMCC) =0.86; *p*-value < 0.05). The maximum fold change detected by the Stratagene was 2.59 compared to 2.28 by the 96.96 dynamic array (Fig. 4).

### Grapevine protection induced by BTH and FOS treatments

**Fig. 5:**
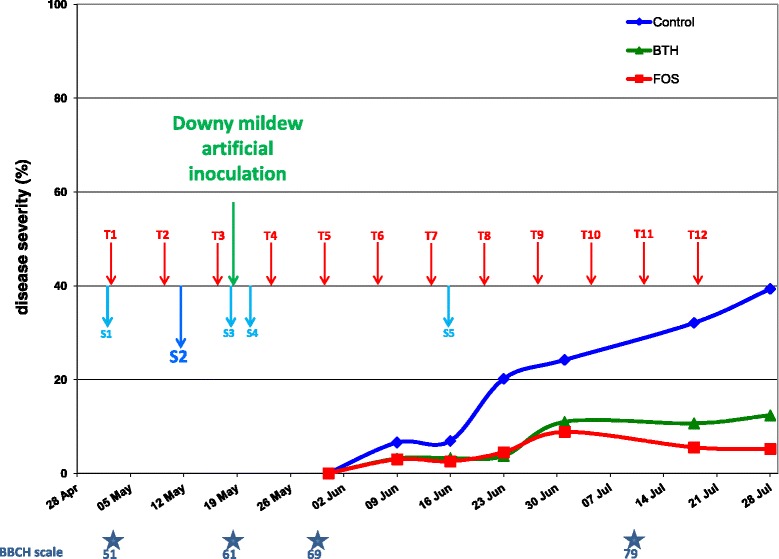
Efficacy of potential defense inducers on leaves against grapevine downy mildew (*P. viticola*). Tests were carried out on a randomized block design with 4 blocks and 3 grapevine plants per block of Cabernet Sauvignon. Three modalities were studied: untreated, treated every week with 1Kg Ha-1 of active ingredient of BTH (Acibenzolar-S-methyl 50%, Bion® 50WG, Syngenta) and treated with 2.5 Kg Ha-1 of active ingredient of fosetyl aluminum (Fosetyl-Al 80%, Aliette®Flash, Bayer). Treatments were carried out between 3rd May and 19th July 2011 (12 treatments, T1 to T12 and *red arrows*) and with artificial inoculation performed on 19th May 2011 (*green arrow*). Disease severity was assessed 5 times between 9th June 2011 (after 5 treatments and 3 weeks after artificial inoculation) and 28th July by assessing the extent of attack on 30 leaves per block during the season. Leaves were sampled throughout the season: before any treatment to check the homogeneity of the parcel (S1), 48 h after the second treatment (S2), 48 h after the third treatment and just before artificial inoculation (S3), 48 h after artificial inoculation (S4), then later, 48 h after the seventh treatment (S5)

**Fig. 6:**
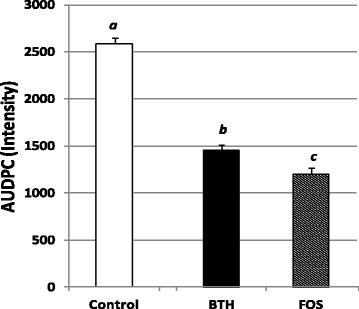
Area under the disease progress curve (AUDPC). Data from disease progression curves from 9th June to 28th July 2011 were transformed in a single value by a formula developed by Simko and Piepho [48], the area under the disease progress curve (AUDPC). Values are means ± SD of AUDPC obtained

The effect of BTH and FOS on downy mildew was evaluated. The mode of action of BTH is only through stimulation of plant defenses [17, 27], while that of FOS is more complex with direct and indirect effects [9]. Previous studies showed that this complex mode depends on the dose applied by soil drenching [33], with an indirect effect at low dose (<10 mM) and a direct effect at high dose (>50 mM). In our study, FOS was applied on grapevine foliage at the authorized dose (2.5 kG Ha^−1^ corresponding to 7.05 mM) for which a direct action has already been shown leading to an inhibition of 87.5% of downy mildew spores germination at a dose 5 times lower (1.13 mM) [27]. Figure 5 shows that treatment of grapevine leaves with BTH and FOS in field conditions led to a significant reduction in downy mildew symptoms compared to untreated control leaves, with a better efficiency of FOS. The severity of grapevine downy mildew (*Plasmopara viticola*) in FOS and BTH-treated blocks at the end of 28th July were 85 and 70% lower than on untreated blocks, respectively (5.2% ± 1.6 and 12.4% ± 2.5 of downy mildew severity respectively compared to 39.3% ± 2.8 on untreated control). Area Under Disease Progress Curves (AUDPC) [48], which summarize repeated data such as the change in intensity of an epidemic as a unique value (AUDPC), were 55 and 45% lower than in untreated controls, respectively (Fig. 6).

As we observed in preliminary trials, significant effects with this treatment program were observed on the grapevine physiology (spilled flowering and ripening late, data not shown), analyses of yield and fruit quality were made at harvest carried out the 11th October 2011 (weight of harvested bunches, berry weight and pH, acidity and sugar content of the must, Table 3). BTH and FOS had a significant protective effect on grapevine leaves (5.2% ± 1.6 and 12.4% ± 2.5 of downy mildew severity respectively compared to 39.3% ± 2.8 on untreated control) and on grape berries (2.82% ± 0.83 and 2.54% ± 0.67 of downy mildew severity respectively compared to 38.8% ± 2.35 on untreated control, data not shown) and the harvest was 3.3 times higher in the treated vines (1637 ± 280 g with 17.5 ± 2.9 clusters per stock) than in the untreated vines (496 ± 165 g with 8.5 ± 2.3 clusters by stock). The grapes harvested from plants treated with BTH were 2 times smaller than those harvested from plants treated with FOS but identical to grapes harvested in the untreated plants (163.4 ± 27.3 g and 336.5 ± 33.4 g per cluster, respectively) with berries 1.5 times smaller than those of FOS-treated and untreated plants (0.97 ± 0.03 g 1.46 ± 0.02 g by berry, respectively) (Table 3). No difference was observed in pH, acidity and sugar in sugar content (Table 3).

**Table 3:** Assessment of the yield and the fruit quality at the harvest carried out on 11th October 2011

	mean weight harvested by stock (g)	cluster mean weight (g)	berry mean weight (g)	sugar content (g/L)	Acidity	pH
Control untreated	496,7 ± 164,6 ***a***	133,3 ± 38,0 ***a***	1,46 ± 0,03 ***b***	175,8 ± 0,7 ***a***	4,2 ± 0,04 ***a***	3,3 ± 0,04 ***a***
BTH treated	1480,0 ± 296,1 ***b***	193,6 ± 21,4 ***a***	0,97 ± 0,03 ***a***	180,9 ± 2,2 ***a***	4,0 ± 0,13 ***a***	3,4 ± 0,13 ***a***
FOS treated	1794,2 ± 262,9 ***b***	336,5 ± 33,1 ***b***	1,47 ± 0,01 ***b***	174,9 ± 0,2 ***a***	4,2 ± 0,02 ***a***	3,2 ± 0,02 ***a***

Values with the same letter are not statistically different at a threshold of 0.05%

We are quite aware that the field test presented in this study will never be used by the profession: it only served as a example to validate this tool for assessing grapevine defense status in the natural environment, and in no case to develop this alternative strategy (elicitor used).

The molecular tool used for several years in previous tests (“BioMolChem” chip or “qPFD” chip) was limited when we wanted to test the grapevine defense status in vineyard. We needed to increase the throughput of gene expression analyses.

We developed the Fluidgm tool, and in this paper, we underline its power, which has a throughput 60–70 times higher and uses amounts of cDNA 70–150 times smaller than with conventional qPCR assays. Only the second sample in this field trial was analyzed by the two technologies.

### Defense-related gene expressions in elicited grapevine leaves

**Fig. 7:**
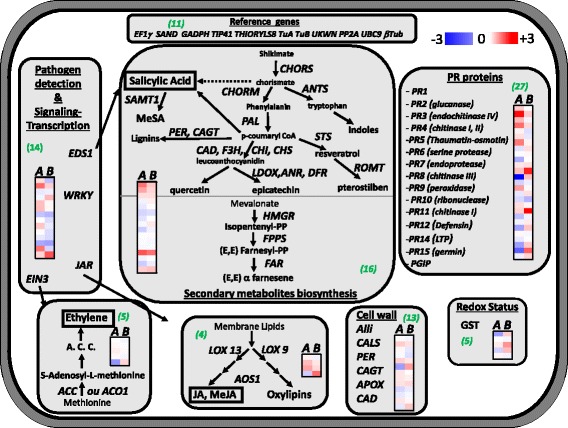
“Heatmap” representing relative expression of genes in Log_2_ transformations. Expression levels of 85 defense-related genes involved in pathogen detection-signaling-transcription (*n* = 10), in secondary metabolite biosynthesis (*n* = 26), coding for PR-proteins (*n* = 29), involved in cell wall reinforcement (*n* = 13), in oxylipins/JA and ET biosynthesis (*n* = 3) and redox status (*n* = 5) were assessed using a relative method with multiple-gene normalization (11 genes) in grapevine leaves treated with BTH (**a**) or FOS (**b**) compared to untreated leaves. Values are means ± SD of three independent biological replicates. The color gradient leading to blue for genes repressed (Log_2_(RE) < 0), to red for genes over-expressed (Log_2_(RE) > 0) and white for genes exhibiting no modification in their expression (Log_2_(RE) = 0). Numbers in brackets: number of genes involved in the function

As the expected action of these potentially eliciting products is rather preventive than curative and because preliminary studies [26] showed that the pathogen diverts the plant metabolism in its favor and particular by blocking the deployment of its defenses, we chose a sampling protocol rather early in the season when the period of grapevine sensitivity against downy mildew is the strongest than later after the pathogen inoculation or once the epidemic is in place at a time when there was no real point in tracking the defense status of the vine. The grapevine defense reaction was analyzed 48 h after a second elicitor treatment applied in the vineyard. Twelve leaves at a similar stage were taken for each modality 2 days after the second treatment (see Methods, Fig. 5). Among the genes involved in pathogen detection- signaling- transcription, BTH induced (Relative Expression (RE) > 1.2) the genes involved in the SA and ET pathways, with the SA-dependent (*EDS1b*) gene, SA-methyl transferase (*SAMT1*), the enzyme involved in ethylene biosynthesis (*ACO1a*) and a transcription factor (*WRKY2*) (Table 3 and Fig. 7).

In the PR protein gene expressions, BTH treatment triggered the over-expression (*RE* > 1.2) of *PR1* transcripts (*PR1* and *1bis*), glucanases (*PR2* and *GLU*), chitinases (*PR3*, *CHIT3*, *CHIT4* and *PR4*), serine protease (*PR6* and *6bis*) and the repression (*RE* < 0.8) of the other serine protease (*PIN*). BTH treatment also led to the repression of subtilisin-like endoproteases (*PR7-7bis*), ribonuclease-like (*PR10*), defensin-like (*PR12*) and the germin-like protein- oxalate oxidase genes (*PR15-15bis*) (Table 4 and Fig. 7). Among the genes involved in cell wall reinforcement, BTH led to the up-regulation (*RE* > 1.2) of coniferyl alcohol glucosyl transferase (*CAGT*) and lignin forming peroxidase (*PER*) and to the repression (*RE* < 0.8) of the other *CAGT2*. BTH also led to the up-regulation (*RE* > 1.2) of allinase (Alli2) which is involved in the production of volatile compounds.

**Table 4:** Relative expression of defense-related genes in “NeoViGen96” chip

*Genes*	Control	BTH-treated	FOS-treated
*PR1*	1.15 ± 0.69	1.52 ± 0.34	1.00 ± 1.05
*PR1 bis*	1.00 ± 0.09	7.37 ± 2.81	1.51 ± 0.69
*GLU*	1.01 ± 0.21	1.77 ± 0.66	0.92 ± 0.09
*PR2*	1.01 ± 0.12	3.31 ± 1.32	1.39 ± 0.64
*PR3*	1.01 ± 0.18	1.12 ± 0.23	0.77 ± 0.12
*CHIT4a*	1.01 ± 0.14	1.28 ± 0.13	0.82 ± 0.17
*PR4*	1.01 ± 0.20	2.99 ± 0.98	1.13 ± 0.36
*PR4bis*	1.01 ± 0.20	0.80 ± 0.29	1.34 ± 0.99
*PR5bis*	1.00 ± 0.11	0.98 ± 0.18	1.08 ± 0.21
*PIN*	1.00 ± 0.07	0.51 ± 0.21	1.01 ± 0.42
*PR6*	1.04 ± 0.32	1.23 ± 0.44	1.30 ± 0.18
*PR6bis*	2.74 ± 3.94	1.67 ± 1.07	6.31 ± 10.45
*PR7*	1.24 ± 0.94	0.01 ± 0.00	1.64 ± 2.43
*PR7 bis*	1.01 ± 0.20	0.69 ± 0.05	0.88 ± 0.08
*CHIT3*	1.04 ± 0.34	2.28 ± 0.93	1.07 ± 0.42
*PR8*	1.04 ± 0.39	0.82 ± 0.09	1.32 ± 0.97
*POX*	1.06 ± 0.45	0.73 ± 0.27	0.55 ± 0.25
*PR9-b*	1.25 ± 1.04	1.45 ± 1.38	17.97 ± 27.30
*PR10*	1.00 ± 0.05	0.59 ± 0.03	0.91 ± 0.35
*PR11*	1.00 ± 0.12	0.91 ± 0.28	1.18 ± 0.63
*PR12*	1.01 ± 0.21	0.47 ± 0.11	1.61 ± 1.17
*PR14*	1.02 ± 0.25	1.07 ± 0.17	0.93 ± 0.09
*PR 14bis*	1.02 ± 0.23	0.83 ± 0.06	0.98 ± 0.18
*PR15*	1.19 ± 0.69	0.46 ± 0.15	1.55 ± 1.45
*PR15bis*	1.39 ± 1.11	0.27 ± 0.23	3.72 ± 6.41
*PGIP*	1.03 ± 0.30	0.89 ± 0.21	1.43 ± 0.56
*PAL*	1.00 ± 0.06	3.17 ± 0.24	1.56 ± 0.35
*STS*	1.01 ± 0.17	2.11 ± 0.43	1.43 ± 0.18
*ROMT*	1.00 ± 0.10	1.32 ± 0.65	1.34 ± 0.65
*CHS*	1.00 ± 0.11	0.90 ± 0.21	1.14 ± 0.11
*CHS2*	1.00 ± 0.09	0.83 ± 0.14	1.03 ± 0.15
*CHI*	1.01 ± 0.13	0.71 ± 0.17	0.77 ± 0.15
*CHI2*	1.01 ± 0.14	0.80 ± 0.13	1.00 ± 0.08
*DFR*	1.02 ± 0.24	0.95 ± 0.17	1.21 ± 0.32
*LDOX*	1.01 ± 0.14	0.68 ± 0.09	0.90 ± 0.17
*PPO*	1.08 ± 0.46	0.99 ± 0.19	1.05 ± 0.37
*HMGR*	1.01 ± 0.16	0.97 ± 0.09	0.94 ± 0.10
*FPPS*	1.01 ± 0.17	0.95 ± 0.17	0.96 ± 0.16
*FAR*	1.02 ± 0.22	4.08 ± 2.97	3.20 ± 3.84
*FAR2*	1.03 ± 0.29	0.91 ± 0.20	1.00 ± 0.28
*F3H*	1.00 ± 0.11	0.67 ± 0.12	0.87 ± 0.21
*HSR203J*	1.06 ± 0.40	1.50 ± 0.34	1.66 ± 0.75
*ANTS*	1.01 ± 0.13	1.03 ± 0.10	0.92 ± 0.17
*CHORM*	1.00 ± 0.10	1.07 ± 0.24	0.98 ± 0.11
*CHORM2*	1.00 ± 0.07	1.16 ± 0.30	1.06 ± 0.11
*CHORS*	1.00 ± 0.04	1.07 ± 0.10	0.92 ± 0.06
*CHORS2*	1.10 ± 0.51	0.42 ± 0.03	1.43 ± 1.81
*GST1*	1.00 ± 0.05	0.45 ± 0.26	1.50 ± 0.92
*GST2*	1.00 ± 0.09	0.54 ± 0.11	0.93 ± 0.22
*GST3*	1.08 ± 0.55	1.18 ± 0.20	2.40 ± 2.89
*GST4*	1.02 ± 0.23	0.69 ± 0.22	1.74 ± 0.07
*GST5*	1.01 ± 0.17	1.08 ± 0.37	0.91 ± 0.23
*LOX2*	1.03 ± 0.29	1.13 ± 0.56	0.90 ± 0.08
*LOX9*	1.00 ± 0.11	1.75 ± 0.17	1.23 ± 0.21
*LOX3*	1.01 ± 0.20	2.15 ± 0.26	1.38 ± 0.34
*LOX4*	1.05 ± 0.37	0.66 ± 0.27	3.35 ± 4.80
*Alli*	1.00 ± 0.02	1.04 ± 0.10	0.92 ± 0.13
*Alli2*	1.02 ± 0.28	1.52 ± 0.12	1.29 ± 0.18
*APOX*	1.00 ± 0.07	1.08 ± 0.27	0.82 ± 0.11
*APOX2*	1.01 ± 0.21	0.96 ± 0.18	0.76 ± 0.14
*CAGT*	1.07 ± 0.42	1.47 ± 0.45	1.22 ± 0.66
*CAGT2*	1.12 ± 0.68	0.45 ± 0.08	2.20 ± 2.92
*CALS*	1.01 ± 0.16	1.22 ± 0.12	1.03 ± 0.27
*CALS2*	1.04 ± 0.33	1.23 ± 0.20	1.15 ± 0.11
*CALS3*	1.06 ± 0.46	1.27 ± 0.65	1.38 ± 0.28
*PER*	1.01 ± 0.13	1.33 ± 0.06	1.10 ± 0.21
*PECT1*	1.17 ± 0.83	0.72 ± 0.44	0.85 ± 0.32
*PECT2*	1.00 ± 0.09	0.84 ± 0.08	0.94 ± 0.09
*CAD*	1.01 ± 0.16	1.24 ± 0.03	1.08 ± 0.18
*CAD2*	1.08 ± 0.53	0.83 ± 0.15	1.60 ± 1.51
*EDS1a*	1.00 ± 0.06	1.33 ± 0.32	0.59 ± 0.12
*EDS1b*	1.00 ± 0.04	3.36 ± 0.44	1.50 ± 0.60
*EDS1c*	1.01 ± 0.14	0.83 ± 0.08	0.88 ± 0.19
*WRKY1*	1.01 ± 0.19	0.92 ± 0.20	1.80 ± 2.12
*WRKY2*	1.00 ± 0.08	1.69 ± 0.09	1.08 ± 0.32
*JAR*	1.04 ± 0.32	1.73 ± 0.84	1.07 ± 0.18
*JAR2*	1.01 ± 0.20	0.61 ± 0.06	0.55 ± 0.19
*ACO1*	1.01 ± 0.13	1.00 ± 0.16	0.75 ± 0.05
*ACO1b*	1.02 ± 0.24	1.58 ± 0.10	0.85 ± 0.25
*ACC*	1.01 ± 0.15	1.03 ± 0.23	0.74 ± 0.03
*EIN3*	1.01 ± 0.16	0.83 ± 0.11	0.73 ± 0.06
*EIN3bis*	1.01 ± 0.15	0.50 ± 0.11	0.46 ± 0.12
*SAMT1*	1.01 ± 0.20	3.10 ± 1.60	0.71 ± 0.10
*AOS1*	1.52 ± 1.14	1.41 ± 0.63	4.16 ± 5.17

Results also showed the differential expression of genes involved in the pathways of secondary metabolites, indoles and ET/JA (Table 4 and Fig. 7), with the over-expression of genes involved in stilbene biosynthesis (*PAL*, *STS*, *ROMT*), in isoprenoid biosynthesis (*FAR*) and in oxylipin/JA biosynthesis (*LOX9* and *LOX3*) and the repression of genes involved in flavonoid biosynthesis (*CHI* and *CHI2*, *LDOX* and *F3H*), in the indole pathway (*CHORS2*) and in redox status (*GST1* and *GST4*).

BTH treatment induced the modulation of 14.1% of the studied genes, 58.3% of them being up-regulated with significant over-expression of genes coding for *PR1-1bis*, *PR2, PAL* and *STS* and significant repression of gene coding for *PR7bis* and *LDOX* and *F3H* (Table 5).

**Table 5:** Relative expression of defense-related genes that were significantly induced (*bold and italic*) or repressed (*italic and underlined*) in leaves treated with BTH and FOS in comparison with untreated controls sampled 48 h after the second treatement (S2) at the threshold of 0.05%

	BTH	FOS
*PR1 bis*	***7.37***	-
*PR2*	***3.31***	-
*PR7 bis*	*0.69*	*0.88*
*PAL*	***3.17***	***1.56***
*STS*	***2.11***	-
*LDOX*	*0.68*	-
*F3H*	*0.67*	-
*GST2*	-	*0.93*
*GST4*	-	***1.74***
*LOX9*	***1.75***	-
*EDS1b*	***3.36***	-
*JAR2*	*0.61*	*0.55*
*ACO1b*	***1.58***	-
*EIN3*	-	*0.73*
*EIN3bis*	*0.50*	*0.46*
*% of differentiated genes*	***14.1***	***8.2***
*Relative % of up-regulated genes*	***58.3***	***25***

All of these results corroborated our previous study showing that BTH enhances resistance against *Plasmopara viticola* by up-regulating the PR proteins (*PR1*, *PR2* and *PR3*). The most induced gene was *PR1* which is usually reported to be a typical marker of SAR [49]. A rapid over-expression of three genes coding for key enzymes was observed in the phenylpropanoid pathway (*PAL* and *STS*) and in the SA biosynthesis pathway (*PAL*). One *GST* gene coding for an enzyme involved in the redox status of the plant and an *ACC* gene involved in the ethylene pathway were also strongly up-regulated. [26] The same defense response pathway may therefore be observed in grapevine leaves in the vineyard to that observed in laboratory conditions.

Upon FOS treatment, only the *EDS1a*, *ACO1a* genes were significantly up-regulated and a significant repression of an EIN3-dependent regulation of plant ethylene hormone signaling (*EIN3bis*) and genes coding for important components of the resistance gene JA-dependent (*JAR2)* was observed.

The expression level of defense-related genes in FOS-treated leaves was less intense with only 8.2% of differentiated genes, 25% of them being up-regulated. FOS treatment induced a less strong over-expression of *PR1*-*PR2* (significant in BTH-treated but not in FOS-treated) and the repression of *PR3* while it was over-expressed with BTH, and a less strong repression of *PR7bis*. Genes encoding *PR7*- *PR12* and *PR15* in BTH-treated leaves were repressed while they were over-expressed with FOS treatment. Two major genes in the biosynthetic pathway of phenylpropanoids (*PAL* and *STS*) were significantly over-expressed in leaves treated with BTH while only *PAL,* which is also involved in the SA pathway, was differentially over-expressed in FOS-treated leaves, with expression levels two-fold and five-fold higher in BTH-treated leaves than in FOS-treated ones and untreated controls, respectively. Similarly, two major genes of the biosynthetic pathway of stilbene (*PAL* and *STS*) were strongly linked to resistance induced by BTH.

These results are in agreement with data obtained in previous experiments conducted in controlled laboratory conditions [16, 17] and also in another where *PAL* and *STS* were reported to be major genes in the resistance of *Vitis vinifera* [50].

**Fig. 8:**
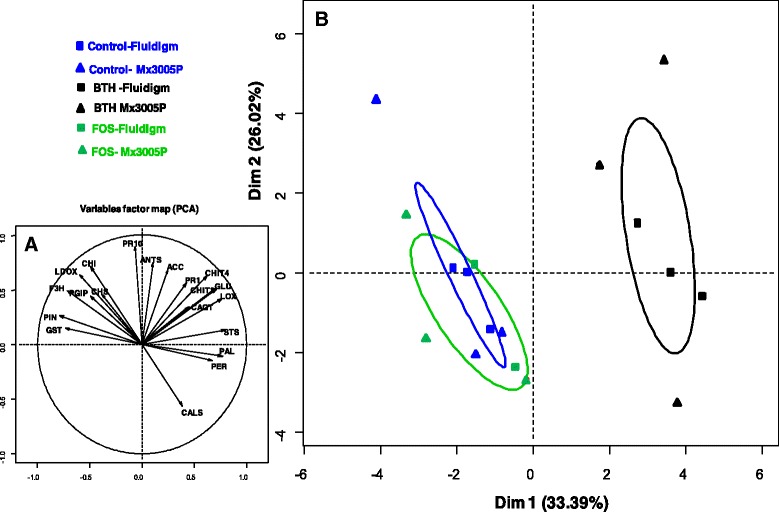
Principal component analysis and cluster of genes differentially expressed. PCA of 24 *Vitis vinifera* defense-related gene expression data sets for visualizing observations in a 2-dimensional space in order to identify uniform or atypical groups of observations. **a** Projection on the standard unit circle of the quantitative variables (genes): **b** The two major principal components explaining ~ 60% of the expression variance plotted for 18 samples. Gene expression data were obtained by microfluidic dynamic array (*squared plot*) or by the Stratagene Mx3005P system (*triangular plot*). The different groups are indicated by different colors (*blue*: control untreated; *black*: BTH-treated; and *green*: FOS-treated)

Principal Component Analysis (PCA) was used to summarize the ways in which gene defense responses vary after elicitation. Most of the variance in the defense expression dataset was contained in the first two principal components which captured ~ 60% of the total variability (Additional file 3). Results of PCA based on the subset of the 24 *Vitis vinifera* defense-related genes common to the two technologies (Tables 1 and 2) summarized the 18 samples projected on the two principal components (Fig. 8). Statistical similarities analysis (ANOSIM) showed no significant difference between gene-expression analyses performed with Fluidigm microfluidics dynamic arrays (squared samples) and the Stratagene Mx3005P qPCR system (triangular samples) (*p* = 0.95). The axis 1 discriminates BTH- treated modalities treated of all other modalities (control and FOS-treated), marked by a significant over expression of *STS*-*PAL*-*LOX*-*GLU*-*CHIT3*-*PER* and *CHIT4* genes and significant repression *CHI* -*PGIP*-*LDOX*-*F3H*-*GST* and *PIN* genes in these samples. By cons, although variables *Pr10*-*ANTS*-*CHI*-*ACC*-*CHIT4*-*LDOX*-*PR1*-*CHIT3*-*GLU* and *CALS* genes contribute significantly to the axis 2, no particular modality was significantly correlated with this axe 2 (Fig. 8). Confidence ellipses around the categories of treatments revealed two significant clusters: Cluster A with control and FOS-treated samples and Cluster B only with BTH-treated samples.

Gene expression analyses made on leaves sampled 48 h after downy mildew inoculation (S4, Fig. 5) showed that the BTH treatment continued to modulate 33.3% of the studied genes (*N* = 24 in Stratagene system), 87% of them being up-regulated (data not shown). The significant efficacy of the BTH treatment seems to be due to the stimulation of grapevine defenses, although the level of protection it provides is lower than with FOS.

On the other hand, the grapevine defense responses to the downy mildew attack in FOS-treated plants resulted in a modulation of 71% genes studied and 83% were significantly repressed (data not shown). These results are consistent with previous studies [26], where the pathogen modulated the plant defenses in its favor, including suppressing the defense genes. At the concentration used (2.5 kG Ha-1), the FOS efficiency observed in this trial is probably mostly due to a fungicide effect in view of the low level of defense-related gene expression compared to the untreated control.

## Conclusions

In this paper we report the development of a new high-throughput Q-PCR methodology adapted to monitor grape defense responses. With the Stratagene system and the “BioMolChem” chip, 2 samples were analyzed with 24 primer sets (48 data) by real-time RT-qPCR. The time to run one plate in this system is 1:30 h. With the Biomark HD system, it is possible to obtain 9216 data (96 samples matched with 96 primer sets) in 4 h. This new flexible method has a throughput 60–70 times higher and uses amounts of cDNA 70–150 times smaller. Furthermore, the samples and reagents used are approximately 6 times cheaper than with conventional assays.

The “NeoViGen96” chip allowed us to demonstrate the defense-stimulating effect of BTH in the vineyard, leading to a partial but significant protection against downy mildew. With FOS, the grapevine protection obtained against downy mildew in the vineyard could not be explained by weak elicitor activity so this suggests that it has a strong fungicide action in our hands.

It is now possible to obtain better and easier understanding of grapevine responses to elicitation in the field. The potential of elicitors can be exploited by combining them in innovative pest management programs in association or in alternation with conventional fungicides in order to reduce the use of fungicides.

## Methods

### Plant materials and treatments

Experiments were carried out in the experimental vineyard of Couhins which covers 45 ha and is located near Bordeaux (Pessac-Léognan appellation). The soil is composed of a layer of clay on limestone that is very well suited to Cabernet Sauvignon grafted onto the Fercal rootstock. Double Guyot management provides good leaf distribution and spread.

Tests were carried out on a randomized block design with 4 blocks and 3 grapevine plants per block of Cabernet Sauvignon. Three conditions were studied: untreated, treated every week with 1Kg Ha^−1^ of active ingredient of BTH (Acibenzolar-S-methyl 50%, Bion® 50WG, Syngenta) and treated with 2.5 Kg Ha^−1^ of active ingredient of fosetyl aluminum (Fosetyl-Al 80%, Aliette®Flash, Bayer).

The treatments began on 3rd May 2011 at the phenological stage 13–14 on the BBCH scale (3–4 leaves unfolded) and were stopped on 19th July 2011at stage 79 (fruits and berries have reached final size) after 12 treatments. *Plasmopara viticola* was artificially inoculated on 19th May 2011 at stage 55 (inflorescence swelling, flowers closely pressed together), 24 h after the fourth treatment, by spraying 6 leaves with a solution of sporangia (25 000–45 000 sporangia mL^−1^). Twelve leaves at a similar stage were taken for each assay on 12th May 2011 (2 days after the second treatment on 10th May), divided into three biological repetitions of four leaves and were frozen at −80 °C until use for molecular analysis. Five leaf samplings were performed throughout the season: before treatment to check the homogeneity of the parcel (S1), 48 h after the second treatment (S2), 48 h after the third treatment and just before artificial inoculation (S3), 48 h after artificial inoculation (S4), then later, 48 h after the seventh treatment (S5). In this article we report only the results obtained with the second sampling, prior to P. viticola inoculation, which was the only one to be analyzed with both technologies (Stratagene and BiomarkHD).

### Field study of fosetyl aluminum and BTH effects on downy mildew disease

The progress of the disease was observed several times throughout the epidemic. The downy mildew disease severity (average percentage of attack) was assessed 6 times, beginning on 9th June after 6 treatments, 4 weeks after artificial inoculation, and around 18th July (Fig. 5) by assessing the level of attack of 30 leaves per block according to the CEB method No. 007: Method of practical effectiveness for fungicide tests designed to fight against downy mildew (Plasmopara viticola (B. C) Berl and Tomi).

Data from a disease progress curve were transformed into a single value by a formula developed by Simko and Piepho [48] which calculates the area under the disease progress curve (AUDPC):

$$ AUDPC={\displaystyle \sum_{\left(i=1\right)}^{\left(n-1\right)}\frac{{\mathrm{y}}_{\mathrm{i}}+{\mathrm{y}}_{\mathrm{i}+1}}{2}\times \left({\mathrm{t}}_{\mathrm{i}+1}\hbox{-} {\mathrm{t}}_{\mathrm{i}}\right)} $$

All clusters of each experimental block were collected on 11th October 2011, counted and weighed to assess the yield. This allowed the evaluation of the average number of bunches produced per vine stock as well as the average weight of bunches. In addition, berries randomly picked by modality were weighed to assess the average weight of a berry.

To assess the harvest quality, 3 batches of 3 clusters per modality were crushed and the sugar content, pH and acidity of must obtained were measured. The pH was measured with a pHmeter, the acidity was determined by the volume of NaOH (0.1 N) required to adjust the pH of the must to 7 and the sugar content was measured with a Brix refractometer (a Brix degree correspond to 1% of sucrose in the solution).

### PCR primer pairs

The most recent molecular information available for designing a Biomark assay can provide valuable results in terms of pathways involved in the response of the plant to the pathogen.

The mRNA sequences of the genes studied were taken from the National Center for Biotechnology Information (NCBI) Gene Database or from the Kyoto Encyclopedia of Genes and Genomes (KEGG) pathway Database with *Vitis vinifera* (Wine grape) as reference genome. For primer design, the Primer3 free software (http://bioinfo.ut.ee/primer3-0.4.0/) was used.

The specificity of the primer pairs was checked by melting curve analysis and gel electrophoresis of the amplified product (data not shown). PCR efficiencies of the assays were determined with a 5-point dilution series of a pool of samples from the experiment in qPCR triplicates, in agreement with Bustin et al.[51]. The gene names and symbols, their corresponding accession numbers and the primer sequences that were used are listed in (Tables 1 and 2).

### RNA extraction and reverse transcription

RNA extraction was performed according to the protocol described by Reid et al. [52] from frozen leaves of three biological replicates per treatment (untreated, BTH and FOS) stored at 80 °C. A total of 9 samples formed by 4 leaves were extracted. After grinding in liquid nitrogen, leaf powder was added to an extraction buffer (20 g.mL −1) preheated to 56 °C (300 mM Tris HCl, pH 8.0, 25 mM EDTA, 2 mM NaCl 2% CTAB, 2% poly -vinyl poly-pyrrolidone (PVPP), 0.05% spermidine trihydrochloride and 2% β-mercaptoethanol added extemporaneously). The mixture was stirred vigorously and incubated in a water bath at 56 °C for 10 min under regular stirring. An equal volume of chloroform: isoamyl alcohol (24:2, v/v) was added and then centrifuged at 3500 g for 15 min at 4 °C.

The following RNA extraction steps were conducted using the Spectrum™ Plant Total RNA Kit protocol: RNA was captured onto a binding column using a unique binding solution, which effectively prevents polysaccharides as well as genomic DNA from clogging the column. Residual impurities and the most residual genomic DNA were removed by DNase treatment according to the manufacturer’s procedure and with wash solutions. Purified RNAs were eluted in RNase-free water. The amounts of RNA obtained were measured at 260 nm and 280 nm by spectrometry (NanoDrop 1000 Spectrophotometer, France). RNA integrity was assessed either by electrophoresis on an agarose gel or by passage over a Bioanalyzer (Agilent technology, France). RT-qPCR was conducted according to the MIQE (minimum information for publication of quantitative real-time PCR experiments) guidelines [51].

Ten micrograms were reverse-transcribed using 2 μM oligo-d(T)_15_, ribonuclease inhibitor and M-MLV reverse transcriptase (Promega, France) according to the manufacturer’s instructions in final volume of 900 μl with a final concentration between 70 and 150 ng μL^−1^. The cDNAs obtained were then stored at −20 °C. Each data point is based on three independent biological replicates (biological and non-technical replicates).

### Real-time qPCR with Stratagene Mx3005P system

The expression of the selected genes was assessed by using a Stratagene Mx3005P qPCR system (Agilent technologies) with SYBR Green to detect dsDNA synthesis. For each reaction, 1 μL of each primer at 1 μM and 7 μL of 2 × MESA BLUE qPCR MasterMix Plus for SYBR® Assay Low ROX (Eurogentec) including Hot start DNA polymerase, dNTP and MgCl_2_ and 5 μL of cDNAs, were used according to the manufacturer’s instructions (350–750 ng of cDNA by well). Each PCR reaction was done in duplicate. The PCR was performed at 94 °C for 15 min, followed by 40 cycles at 95 °C for 10 s, 55 °C for 20 s and 72 °C for 20 s. Data were analyzed with MxPro QPCR Software (Agilent technologies) as the cycle of quantification (Cq), where the fluorescence signal of the amplified DNA intersected with the background noise. For each gene and for each modality, a mean Cq value was obtained.

The Cq values >30 were regarded as invalid and treated as missing data. ΔCq was obtained by subtracting the reference gene (EF1γ) Cq mean from the target gene Cq value. The Relative Expression (RE) was calculated with the 2 ^-ΔΔCq^ method for every sample where ΔΔCq was the ΔCq difference between two samples.

### Expression analysis using 96.96 dynamic arrays

Eleven genes were included in the qPCR array to select the endogenous reference genes (Table 2). A 96.96 Dynamic Array IFC plate was also used to analyze the expression levels of the selected genes. The cDNA was first pre-amplified before being analyzed by qPCR with Fluidigm technology: cDNAs were diluted to ~ 5 ng μl^−1^ and pre-amplification was carried out by adding the reaction mixture containing 96 pairs of primers (primers pool, 50 mM) and the TaqMan PreAmp Master Mix (1:2, Applied Biosystems) with 14 cycles of 95 °C for 15 s and 60 °C for 4 min. The pre-amplified cDNA was diluted with TE buffer (1:5) and used for qPCR analysis in a reaction mixture containing TaqMan Gene Expression Master Mix (Applied Biosystems), DNA Binding Dye Sample Loading Reagent (Fluidigm, Issy les Moulineaux, France) and EvaGreen (Interchim, Montlucon, France).

Real-time qPCR was performed using a BioMark ^TM^ HD system (Fluidigm Corporation). The 96.96 dynamic array was used for qPCR, according to the manufacturer’s protocol (http://www.fluidigm.com/user-documents). Five μl of mixture were prepared for each sample containing 1x TaqMan Universal Master Mix (without UNG), 1x GE sample loading reagent (Fluidigm PN 85000746) and each diluted pre-amplified cDNA. The loaded chip was placed in the BioMark system for PCR at 95 °C for 10 min, followed by 40 cycles at 95 °C for 15 s and 60 °C for 1 min. The data were analyzed by using real-time PCR BioMark 2.0 Analysis software (Fluidigm Corporation, France) as the cycle of quantification (Cq) and applying the same principle of classical real-time PCR with the Stratagene MX3005P system where the fluorescence signal of the amplified DNA intersected with the background noise.

Cq values >30 were regarded as invalid and treated as missing data. Expression levels were calculated based on a multiple gene normalization method and using the principles and formulas of Vandesompele et al. [47]. The geometric mean of several carefully selected reference genes (Table 2) was used as an accurate normalization factor. The lowest gene stability value (*M* values) indicates genes with the highest gene expression stability. In all the experiments carried out with Fluidigm, all *M* values of the 11 reference genes were collected (15 independent experiments) to obtain a data set sufficient to assess their stability.

The data were analyzed by using real-time PCR BioMark 2.0 software (Fluidigm Corporation) Analysis.

After completion of the run, a melting curve of the amplified products was determined to confirm the specificity of the reactions.

### Statistical analyses

Statistical analysis was performed using the R statistical software (R Development Core Team, 2010). AUDPC data (progress of downy mildew disease) and yield data at the harvest were compared with an analysis of variance (ANOVA) and significant differences were determined by Tukey’s honest significant difference (HSD) test at the level of *P* ≤ 0.05. To model the relationship between gene expressions obtained with the two platforms, a linear regression analysis was conducted and a Pearson correlation was determined at a level of *p*-value ≤ 0.05.

Genes were observed as differentially expressed for a *p*-value <0.05 in rank-based nonparametric multiple comparisons with the “nparcomp” package in the R statistical software. Significant differences were determined by Dunnett’s test at the level of *p* ≤ 0.05.

A mathematical procedure that transforms a number of possibly correlated variables (gene expressions) into a smaller number of uncorrelated variables called principal components was performed to analyze plant defense behavior after elicitation with the “Rcmdr” package and plug-in “FactoMiner” of R statistical software.

An analysis of similarities (ANOSIM) was made to test the difference between the two methods used (Fluidigm *vs* Stratagene) through the use of “vegan” package of R statistical software.

## Additional files

Additional file 1: Original sequences and/or references used to find new candidate genes involved in grapevine defenses [26, 38, 52–63]. (DOCX 42 kb)
Additional file 2: Protein sequence homology/orthology between *Vitis* and *Arabidopsis and/or Malus domestica - Solanum lycopersicum - Nicotania tabaccum. (XLSX 18 kb)*

Additional file 3: Plot of Eigen values of the principal component Most of the variance in defense gene expression data set is contained in the first two principal components with ~ 60% of the total variability. (DOCX 76 kb)
Additional file 4: Relative expression data set of defense-related gene obtained in classical qPCR system (Stratagene) and in Biomark® system analyzed during this study. (XLSX 48 kb)

## Abbreviations

ABA: Abscissic acid
AUDPC: Area under disease progress curve
BBCH-scale: “Biologische **B**undesanstalt, **B**undessortenamt und **CH**emische Industrie”-scale
BTH: S-methyl benzo[1,2,3]thiadiazole-7-carbothioate (Bion, 50WG, Syngenta)
cDNA: Complementary Deoxynucleotidic acid
Cq: Cycle quantification
CTAB: Cetyl trimethylammonium bromide
EDTA: Ethylenediaminetetraacetic acid
ET: Ethylene
FOS: Fosetyl aluminium
HCl: Hydrogen chloride
JA: Jasmonic acid
KEGG: Kyoto encyclopedia of genes and genomes
MAPK: Mitogen-activated protein kinase
MAPKK: MAPK kinase
MAPKKK: MAPKK kinase
MgCl_2_: Magnesium chloride
NaCl: Sodium chloride
NCBI: National Center of Biotechnology Information
NTP: Nucleotide triphosphate
PCA: Principal component analysis
PR-protein: Pathogenesis related protein
PVPP: Poly-vinyl-poly-pyrrolidone
RE: Relative expression
RNA: Ribonucleotidic acid
ROS: Reactive oxygen species
SA: Salicylic acid
SAR: Systemic acquired resistance
SPD: Stimulator of plant defenses
UNG: Uracil N-glycosylase
