# Supplementary material for: High-throughput gene-expression quantification of grapevine defense responses in the field using microfluidic dynamic arrays
Source: BMC Genomics. 2016 Nov 22;17:957. doi: 10.1186/s12864-016-3304-z (PMC5120521; doi:10.1186/s12864-016-3304-z)
Supplement: Additional file 1: — Original sequences and/or references used to find new candidate genes involved in grapevine defenses [26, 38, 52–63]. (DOCX 42 kb) [file 12864_2016_3304_MOESM1_ESM.docx]

**Additional File 1:** **Original sequences and/or references used to find new candidate genes involved in grapevine defenses**

| **Gene Functions** | | **Gene Names** | **Origin sequences** | **References** |
| --- | --- | --- | --- | --- |
| **Reference genes** | γ Chain Elongation factor 1 | ***EF1γ^*^*** | *Vitis vinifera -* AF176496 | Dufour *et al*., 2013 [26] |
|  | Serine/threonine-protein phosphatase 2A | ***PP2A*** | *Arabidopsis Thaliana* - AT1G13320  *Vitis vinifera* | Hong *et al.*, 2010 [53]  This study |
|  | SAND family protein (endocytosis) | ***SAND*** | *Arabidopsis Thaliana* - AT2G28390  *Vitis vinifera* Vvi.6619 | Hong *et al.*, 2010 [53]  This study |
|  | Pentatricopeptide repeat-containing protein | ***Unknown*** | *Arabidopsis Thaliana* - AT1G62930  *Vitis vinifera* XM 002274855 | Hong *et al.*, 2010 [53]  This study |
|  | Ubiquitin Conjugating Enzyme 9 | ***UBC9*** | *Arabidopsis Thaliana* - AT4G27960  *Vitis vinifera* Vvi.6876/3243 | Hong *et al.*, 2010 [53]  This study |
|  | TIP41-like protein | ***TIP41*** | *Arabidopsis Thaliana* - AT4G34270  *Vitis vinifera* Vvi.7091 | Hong *et al.*, 2010 [53]  This study |
|  | Catalytic thioredoxin-like protein 4A | ***THIORYLS8*** | *Arabidopsis Thaliana* - AT5G08290  *Vitis vinifera* Vvi.87954 | Hong *et al.*, 2010 [53]  This study |
|  | α tubulin | ***TuA*** | *Vitis vinifera* | Reid *et al.*, 2006 [52] |
|  | β tubulin | ***βTub*** | *Vitis vinifera* | Reid *et al.*, 2006 [52] |
|  |  | ***TuB*** |  |  |
|  | Glyceraldehyde 3-phosphate dehydrogenase | ***GADPH*** | *Vitis vinifera* | Reid *et al.*, 2006 [52] |
| **PR protein** | PR1 Unknown function | ***PR1^*^*** | *Vitis vinifera -* AJ536326.1 | Dufour *et al*., 2013 [26] |
|  |  | ***PR1 bis*** | *Malus domestica* - AF50797.1  *Vitis vinifera* Vvi.8923 | de Bernonville *et al*., 2014 [38] and INRA patent |
|  | Beta-1,3-glucanase | ***GLU^*^*** | *Vitis vinifera -* AF239617 | Dufour *et al*., 2013 [26] |
|  |  | ***PR2*** | *Malus domestica* - AF494404  *Vitis vinifera* Vvi.142 | de Bernonville *et al*., 2014 [38] and INRA patent |
|  | Endochitinase (Chitinase IV) | ***PR3*** | *Vitis vinifera* Vvi.8171 | Robinson *et al.*, 1997 [54] |
|  |  | ***CHIT4a^*^*** | *Vitis vinifera -* U97521.1  *Specific* | Dufour *et al*., 2013 [26] |
|  | Chitin binding Chitinases type III | ***PR4*** | *Malus domestica* - CN877594 | de Bernonville *et al*., 2014 [38] and INRA patent |
|  |  | ***PR4bis*** |  |  |
|  | Thaumatin-like / Osmotin | ***PR5*** | *Malus domestica* - DQ318214  *Arabidopsis Thaliana* - AT1G2003  *Vitis vinifera* Vvi.244494  Non specific | de Bernonville *et al*., 2014 [38] and INRA patent  This study |
|  |  | ***PR5bis*** | *Arabidopsis Thaliana* - AT1G75800  *Vitis vinifera* Vvi.8525, 9248-244494 | This study |
|  | Proteinase inhibitor | ***PIN^*^*** | *Vitis vinifera -* XM-002284418 | Dufour *et al*., 2013 [26] |
|  |  | ***PR6*** | *Arabidopsis Thaliana* - AT2G38870  *Vitis vinifera* Vvi.143 | Sels *et al.*, 2008 [55]  This study |
|  |  | ***PR6bis*** | *Vitis vinifera* Vvi.12527 | This study |
|  | Subtilisin-like endoprotease | ***PR7*** | *Solanum lycopersicum* - X95270  *Vitis vinifera -* XM-002284065 | Tornero *et al.*, 1996 [56]  This study |
|  |  | ***PR7 bis*** | *Vitis vinifera -* XM-002272733 | This study |
|  | Chitinase type III | ***CHIT3^*^*** | *Vitis vinifera* Vvi.18 | Dufour *et al*., 2013 [26] |
|  |  | ***PR8*** | *Malus domestica* - DQ318214  *Vitis vinifera* Vvi.8699 | de Bernonville *et al*., 2014 [38] and INRA patent  This study |
|  | Lignin-forming peroxidase | ***POX*** | *Arabidopsis Thaliana*  *Vitis vinifera* XM 002274762.1 | This study |
|  |  | ***PER*** | *Arabidopsis Thaliana*  *Vitis vinifera* | This study |
|  |  | ***PR9-b*** | *Vitis vinifera* Vvi.17743  *Arabidopsis Thaliana*  *Malus domestica* - CN928974 | de Bernonville *et al*., 2014 [38] and INRA patent |
|  | Ribonuclease-like | ***PR10^*^*** | *Vitis vinifera Vvi-25055* | Dufour *et al*., 2013 [26] |
|  | Endochitinase class V | ***PR11*** | *Nicotiana tabacum* - X77110  *Vitis vinifera* Vvi.17292 | Ponstein *et al.*, 1994 [57] |
|  | Defensin | ***PR12*** | *Helianthus annuus* - AAM2791  *Vitis vinifera* XM 002274317.1 | Hu *et al.*, 2003 [58]  This study |
|  | Lipid Transfer Protein | ***PR14*** | *Vitis vinifera* Vvi.7391  *Malus domestica* - CV656658 | This study  de Bernonville *et al*., 2014 [38] and INRA patent |
|  |  | ***PR 14bis*** | *Arabidopsis Thaliana* - AT2G38540  *Vitis vinifera* Vvi.7391-7508-9206-25593-24231 | This study |
|  | Germin-like Protein- Oxalate oxidase | ***PR15*** | *Malus domestica* - GO500607 | de Bernonville *et al*., 2014 [38] and INRA patent |
|  |  | ***PR15bis*** |  |  |
|  | Polygalacturonase Inhibiting Protein | ***PGIP^*^*** | *Vitis vinifera* Vvi.9  AF305093.1  *Specific* | Dufour *et al*., 2013 [26] |
| **Secondary metabolites biosynthesis** | Phenylalanine ammonialyase | ***PAL^*^*** | *Vitis vinifera* | Dufour *et al*., 2013 [26] |
|  | Stilbene synthase (resveratrol synthase) | ***STS^*^*** | *Vitis vinifera* | Dufour *et al*., 2013 [26] |
|  | Resveratrol O-methyl-transferases | ***ROMT*** | *Vitis vinifera* | Schmidlin *et al.*, 2008 [59] |
|  | Chalcone Synthase | ***CHS^*^*** | *Vitis vinifera -* X75969.1  Specific | Dufour *et al*., 2013 [26] |
|  |  | ***CHS2*** | *Vitis vinifera,* Vvi.117  Specific  *Malus domestica* - AF494401 | de Bernonville *et al*., 2014 [38] and INRA patent |
|  | Chalcone Isomerase | ***CHI^*^*** | *Vitis vinifera* Vvi.124 | Dufour *et al*., 2013 [26] |
|  |  | ***CHI2*** | *Arabidopsis Thaliana* - AT3G55120  *Vitis vinifera* Vvi.2800 | This study |
|  | Dihydro Flavonol Reductase | ***DFR*** | *Malus domestica* - AF494390  *Vitis vinifera* Vvi.120 | de Bernonville *et al*., 2014 [38] and INRA patent  This study |
|  | Leucoanthocyanidin dioxygenase | ***LDOX^*^*** | *Vitis vinifera* Vvi.127  *Specific* | Dufour *et al*., 2013 [26] |
|  | Polyphenol Oxidase | ***PPO*** | *Malus domestica* - L29450  *Vitis vinifera* Vvi.15027 | de Bernonville *et al*., 2014 [38]  This study |
|  | 3-hydroxy-3-methylglutaryl Coenzyme A reductase class 1 | ***HMGR*** | *Malus domestica* - AY043490  *Vitis vinifera* Vvi.11686 | de Bernonville *et al*., 2014 [38] and INRA patent |
|  | Farnesyl Pyrophosphate Synthase | ***FPPS*** | *Malus domestica* - AY083165  *Vitis vinifera* Vvi.6767 | de Bernonville *et al*., 2014 [38]  INRA patent  This study |
|  | (E,E)-alpha-farnesene synthase | ***FAR*** | *Malus domestica* - EB111255  *Vitis vinifera* Vvi.6767  XM 002281343.1 | de Bernonville *et al*., 2014 [38] and INRA patent |
|  |  | ***FAR2*** | *Arabidopsis thaliana* - AT1G79460  *Vitis vinifera* Vvi.6767 | This study |
|  | Flavanone-3-hydroxylase | ***F3H^*^*** | *Vitis vinifera-* Vvi.12  Specific | This study |
|  |  | ***F3H bis*** | *Arabidopsis Thaliana* - AT5G24530 | This study |
|  | Carboxylesterase | ***HSR-203J*** | *Vitis vinifera* | Bezier *et al.*, 2002 [60] |
| **Indole biosynthesis** | Antranilate Synthase | ***ANTS^*^*** | *Vitis vinifera* XM-002281597 | Dufour *et al*., 2013 [26] |
|  | Chorismate mutase | ***CHORM^*^*** | *Vitis vinifera* Vvi.19406  XM-002283257.1  Specific | Dufour *et al*., 2013 [26] |
|  |  | ***CHORM2*** | *Vitis vinifera* Vvi.14549  XM 002284083.1 | This study |
|  | Chorismate Synthase | ***CHORS^*^*** | *Vitis vinifera* FJ 604855.1, Vvi.7038 | Dufour *et al*., 2013 [26] |
|  |  | ***CHORS2*** | *Arabidopsis thaliana* - AT5G10870 | This study |
| **Redox status** | Glutathione S-transferase | ***GST1^*^*** | GST Tau Non specific  *Arabidopsis thaliana AT-gstu25*  *Vitis vinifera -* AY 156048, Vvi.17667 | Dufour *et al*., 2013 [26] |
|  |  | ***GST2*** | GST Phi Non specific  *Arabidopsis thaliana*  *Vitis vinifera Vvi-25300-AY156049* | This study |
|  |  | ***GST3*** | GST Phi specific  *Arabidopsis thaliana*  *Vitis vinifera* Vvi.17666  XM 002283178 | This study |
|  |  | ***GST4*** | GST Phi Specific  *Arabidopsis thaliana*  *Vitis vinifera* Vvi.574  XM 002271373 | This study |
|  |  | ***GST5*** | *GST Phi specific*  *Vitis vinifera , Vvi.7684-XM 002283173* | This study |
|  | Ascorbate peroxidase | ***APOX*** | *Vitis vinifera* XM 002284731  *Malus domestica* - CN928974 | de Bernonville *et al*., 2014 [38] and INRA patent |
|  |  | ***APOX2*** | *Vitis vinifera*  *Arabidopsis Thaliana* - AT1G7749 | This study |
| **Oxylipines** | Lipoxygenase 13 | ***LOX2.1***  ***LOX2.2*** | *Vitis vinifera* Vvi.1414  *VvLOXA*  XM 002285538  *Malus domestica* - CN941066 | de Bernonville *et al*., 2014 [38] and INRA patent  Podolyan *et al.*, 2010 [61] |
|  |  | ***LOX2.3*** | Specific | Wu *et al.*, 2010 [62] |
|  | Lipoxygenase 9 | ***LOX9^*^*** | *Vitis vinifera* AY 159556  Vvi.17310, VvLOXC  Non specific | Dufour *et al*., 2013 [26] |
| **Cell wall reinforcement** | Alliinase (sulfoxide cysteine biosynthesis) | ***Alli*** | *Malus domestica* - AY347795  *Vitis vinifera* Vvi.16129 | de Bernonville *et al*., 2014 [38] and INRA patent |
|  |  | ***Alli2*** | *Arabidopsis Thaliana* - AT1G34040  *Vitis vinifera* Vvi.16129 | This study |
|  | Glycosyl transferase (Coniferyl alcohol glucosyl transferase) | ***CAGT^*^*** | *Vitis vinifera*  Specific | Dufour *et al*., 2013 [26] |
|  |  | ***CAGT2*** | *Arabidopsis Thaliana* - AT3G16520  *Vitis vinifera*  Ref-seq | This study |
|  | Callose Synthase | ***CALS^*^*** | *Vitis vinifera* | Dufour *et al*., 2013 [26] |
|  |  | ***CALS2*** | *Vitis vinifera* Vvi.6  *Arabidopsis thaliana*  glucan synthase-like 10 | This study |
|  |  | ***CALS3*** | *Malus domestica* - CN496203  *Vitis vinifera* VVi.8175  *Arabidopsis thaliana*  glucan synthase-like 12 | de Bernonville *et al*., 2014 [38] and INRA patent  This study |
|  | Pectin methyl esterase | ***PECT1*** | *Malus domestica* - CV628630  *Vitis vinifera* Vvi.5361  XM 0022755783 | de Bernonville *et al*., 2014 [38] and INRA patent |
|  |  | ***PECT2*** | *Arabidopsis Thaliana* - AT3G10720  *Vitis vinifera* XM 002283905 |  |
|  | Cinnamyl Alcohol Dehydrogenase | ***CAD*** | *Malus domestica* - AF053084  *Vitis vinifera* | de Bernonville *et al*., 2014 [38] and INRA patent |
|  |  | ***CAD2*** | *Arabidopsis Thaliana* - AT1G09480  *Vitis vinifera* XM 002285332.1 | This study |
| **Signaling pathways** | SA Methyl Transferase | ***SAMT1*** | *Vitis vinifera* | This study |
|  | Allene Oxide Synthase | ***AOS1*** | *Arabidopsis Thaliana* - X92510  *Vitis vinifera* | Laudert *et al.*, 1996 [62] |
|  | Lipase 3 / enhanced disease susceptibility 1 | ***EDS1a*** | *Malus domestica* - CN949066 | de Bernonville *et al*., 2014 [38] and INRA patent |
|  |  | ***EDS1b*** | *Arabidopsis Thaliana* - AT3G48080  *Vitis vinifera* Ref-seq  XM-002281059-002281871 | This study |
|  |  | ***EDS1c*** |  |  |
|  | JAR = Jasmonate-Resistant 1  (Jasmonate Amino Synthase) | ***JAR*** | *Vitis vinifera* Vvi.7914  *Malus domestica* - CN879199 | This study  de Bernonville *et al*., 2014 [38] and INRA patent |
|  |  | ***JAR2*** | *Vitis vinifera* Vvi.7914  *Arabidopsis Thaliana* - AT5G54510 | This study |
|  |  | ***JAR3*** | *Vitis vinifera* Vvi.7914  *Arabidopsis Thaliana* - AT2G46310 | This study |
|  | ACCO 1 = 1-aminocyclopropane-1 carboxylic-acid oxidase 1 | ***ACO1*** | *Vitis vinifera* AY 211549,  Vvi.4992-Vvi.41117  *Malus domestica* - AB086888  Specific | de Bernonville *et al*., 2014 [38] and INRA patent |
|  |  | ***ACO1b*** | *Arabidopsis Thaliana* - AT1G05010  *Vitis vinifera* AY 211549,  Vvi.4992-Vvi.41117 | This study |
|  |  | ***ACC^*^*** | *Vitis vinifera* | Dufour *et al*., 2013 [26] |
|  | EIN3-Binding F Box Protein 1 - ubiquitin protein ligase-like | ***EIN3*** | *Vitis vinifera* Vvi.1771  XM 00228590.1  *Malus domestica* - CV082047  Specific | This study  de Bernonville *et al*., 2014 [38] and INRA patent |
|  |  | ***EIN3bis*** | *Vitis vinifera* Vvi.1771  XM 002285213.1  Specific | This study |
|  | Transcription Factors | ***WRKY1*** | *Vitis vinifera* | Marchive et al., 2007 [63] |
|  |  | ***WRKY2*** | *Vitis vinifera* | This study |
