# Supplementary material for: High-throughput gene-expression quantification of grapevine defense responses in the field using microfluidic dynamic arrays
Source: BMC Genomics. 2016 Nov 22;17:957. doi: 10.1186/s12864-016-3304-z (PMC5120521; doi:10.1186/s12864-016-3304-z)
Supplement: Additional file 3: — Plot of Eigen values of the principal component Most of the variance in defense gene expression data set is contained in the first two principal components with ~ 60% of the total variability. (DOCX 76 kb) [file 12864_2016_3304_MOESM3_ESM.docx]

**Additional File 3: Plot of Eigen values of principal component**
